# Off-pump coronary revascularization performed concomitant with non-cardiovascular surgery

**DOI:** 10.1186/1749-8090-8-S1-O179

**Published:** 2013-09-11

**Authors:** JW Han, KH Lee, HY Hwang, KB Kim

**Affiliations:** 1 Adult Cardiac Surgery, Seoul National University Hospital, Seoul, Korea

## Background

A concern about complications secondary to the cardiopulmonary bypass caused reluctance to perform combined cardiac and non-cardiovascular surgery. We studied the patients who underwent off-pump CABG concomitant with non-cardiovascular surgery.

## Methods

Of 2083 patients who underwent isolated off-pump CABG between 1999 and 2012, 91 patients who underwent off-pump CABG concomitant with non-cardiovascular surgery (group 1) were compared with 1991 patients who underwent isolated off-pump CABG (group 2). In group 1, there were 49 malignancies and 42 benigns. Non-cardiovascular surgery included 34 thoracic surgeries (11 bullectomy, 11 pulmonary wedge resection, 5 thymectomy, 4 lobectomy, 1 tracheoesophageal fistulectomy, 1 radical mastectomy, and 1 diaphragm plication), 56 abdominal surgery (24 gastrectomy, 14 cholecystectomy, 7 colectomy, 5 herniorrhaphy, 2 mass excision, 2 nephrectomy, 1 feeding jejunostomy, and 1 perigastric LN biopsy), and 1 below-knee amputation.

## Results

Mean age at operation were 67.8 ± 9.6 years (group 1) and 63.9 years ± 9.5 (group 2), respectively. There were no significant differences in preoperative risk factors, including smoking, hypertension, diabetes mellitus, dyslipidemia, obesity, stroke, left ventricular dysfunction, and chronic renal failure between the 2 groups. Mean number of distal anastomosis was 2.93 ± 1.0 in group 1 and 3.09 ± 0.97 in group 2 (p=0.133). Operative mortality was 1.1% (1/91) in group 1 and 1.3% (26/1991) in group 2, respectively (p=1.000). There were no significant differences in postoperative morbidities, including mediastinitis, re-exploration for bleeding, perioperative myocardial infarction, low cardiac output syndrome, atrial fibrillation, and stroke between the 2 groups.

## Conclusions

Off-pump CABG concomitant with non-cardiovascular surgery was not associated with increased operative mortality and postoperative morbidities.

